# Rapid Diagnostic PCR Assay Method for Species Identification of Mantidis Ootheca (Sangpiaoxiao) Based on Cytochrom C Oxidase I (COI) Barcode Analysis

**DOI:** 10.3390/ijms251810224

**Published:** 2024-09-23

**Authors:** Sumin Noh, Wook Jin Kim, Ji-Min Cha, Goya Choi, Sungyu Yang, Jun-Ho Song, Byeong Cheol Moon

**Affiliations:** 1 Herbal Medicine Resources Research Center, Korea Institute of Oriental Medicine, Naju 58245, Republic of Korea; pureum322@kiom.re.kr (S.N.); ukgene@kiom.re.kr (W.J.K.); lovely08022@gmail.com (J.-M.C.); serparas@kiom.re.kr (G.C.); sgyang81@kiom.re.kr (S.Y.); 2 Department of Biology, Chungbuk National University, Cheongju 28644, Republic of Korea

**Keywords:** Mantidis Ootheca, cytochrome C oxidase subunit I (COI), molecular authentication, sequence-characterized amplified region (SCAR) marker, multiplex polymerase chain reaction

## Abstract

Mantidis Ootheca (sangpiaoxiao), the egg case of the mantis, is a type of insect-derived traditional medicine widely used in East Asia. However, species identification based on egg morphology is challenging, leading to the distribution of counterfeit and adulterated products. The use of inauthentic ingredients can pose serious health risks to consumers. This study aimed to develop PCR markers that can rapidly and accurately differentiate between authentic and counterfeit Mantidis Ootheca. The mitochondrial cytochrome c oxidase I (COI) region was sequenced in thirteen samples from four mantis species: *Tenodera angustipennis*, *Statilia maculata*, *Hierodula patellifera*, and *T. sinensis*. Four sets of SCAR primers were designed based on species-specific nucleotide polymorphisms, and a multiplex SCAR assay was developed by combining all sets of the primers. The sequence-characterized amplified region (SCAR) primers successfully produced amplicons for each target species, even with low-DNA templates or templates containing DNA from multiple samples. No amplification was observed for nontarget species. This study presents a novel approach for identifying authentic Mantidis Ootheca species using DNA-based diagnostic marker assays, which enable rapid and precise species identification. The SCAR assays developed in this study will aid in maintaining quality control and promoting the standardization of commercial Mantidis Ootheca products.

## 1. Introduction

The use of insects and their products in medicine, known as entomotherapy, has been prevalent worldwide for centuries. Entomotherapy is a noteworthy complement and alternative to modern therapies [1,2,3,4,5]. Traditional entomotherapy has been based on empirical evidence for millennia. However, over the past two decades, technological advances have provided scientific evidence for the pharmacological activities of medicinal insects and their products [1,2,6].

Mantidis Ootheca, an insect-derived traditional medicine used in East Asia, refers to the steamed egg case of the mantis, which belongs to the Mantidae family. It is traditionally used to treat disorders of the genitourinary system, such as renal failure, incontinence, spermatorrhea, and leukorrhea [7,8]. In addition to these traditional uses, modern pharmacological studies have revealed that Mantidis Ootheca possesses anti-inflammatory, antioxidative, antiatherosclerotic, antidiuretic, anticancer, and vascular relaxant properties [7,9,10,11,12,13].

The authentic species of Mantidis Ootheca differ slightly among different countries. According to the Korean Herbal Pharmacopoeia, three species belonging to the Mantidae family have been designated as authentic Mantidis Ootheca: *Tenodera angustipennis* (Saussure, 1869) (tribe Polyspilotini), *Statilia maculata* (Thunberg, 1784) (tribe Mantini), and *Hierodula patellifera* (Serville, 1839) (tribe Paramantini) [14]. In contrast, the Pharmacopoeia of the People’s Republic of China have designated the above species as authentic Mantidis Ootheca species [14], except *T. angustipennis*, which has been replaced by *T. sinensis* (Saussure, 1871). Such differences in the original species of Mantidis Ootheca require special attention in mutual trading between countries for its quality control. Another factor that complicates the quality control of Mantidis Ootheca is the difficulty for non-experienced people to identify species based solely on the morphological characteristics of the egg case [15,16,17]. The potential for non-original species to be mistakenly mixed and distributed as medicinal materials poses a significant risk, because there may be pharmacological differences among different species [12,15]. Thus, for quality standardization, it is necessary to develop objective identification methods that can be applied to distinguish between species and to prevent confusion and the misuse of Mantidis Ootheca.

In the past 15 years, molecular techniques such as DNA barcoding have been developed and widely used for the accurate species identification of morphologically controversial taxa [18,19]. However, the conventional “one-by-one DNA barcoding” method that employs Sanger sequencing has limitations—it is expensive, laborious, and sensitive to the quality of the template DNA. Moreover, when universal primers are used to identify species from unknown samples, they also amplify the genes of storage pests, making accurate species identification difficult [15]. To overcome these limitations, DNA-marker-based techniques have been used for rapid, convenient, and accurate species identification. Sequence-characterized amplified regions (SCARs) are codominant markers that are used in PCR with a pair of species-specific oligonucleotide primers that are designed based on species-specific nucleotide polymorphisms [20,21,22]. Several studies have reported the development of DNA markers for the identification of beneficial insect- and animal-derived medicinal materials [23,24,25,26]. SCAR markers are classified based on the type of DNA polymorphism information they are derived from, and among these, SCAR markers designed from DNA barcode sequences are highly reproducible and less sensitive to reaction conditions because of the stability of the DNA barcode regions [22]. The mitochondrial genome contains many useful DNA barcode regions [27,28]. In insects and animals, the mitochondrial cytochrome c oxidase subunit I (COI) has been primarily used as the DNA barcode region, which provides valuable information for species identification [29]. Various studies utilizing the mitochondrial genome for phylogenetic analyses in the order Mantodea have been documented, including research on COI barcode sequences [30,31,32,33].

This study aimed to develop a novel diagnostic method for authenticating Mantidis Ootheca at the species level that involves the use of SCAR markers and a multiplex SCAR assay, which is more reliable, faster, and simpler than existing methods. This study was based on the mitochondrial COI sequences of four mantis species, namely *T. angustipennis*, *S. maculata*, *H. patellifera*, and *T. sinensis*, which are the original species of Mantidis Ootheca in Korea and China. The developed SCAR markers and multiplex SCAR assays were used to determine the authenticity of commercial Mantidis Ootheca products.

## 2. Results

### 2.1. Nucleotide Sequence Analysis

Thirteen COI sequences (658 bp) from the four mantis species (*T. angustipennis*, *S. maculata*, *H. patellifera*, and *T. sinensis*) were used for the statistical analysis. Among these, *T. angustipennis* showed no intraspecific distance, whereas *H. patellifera* showed the highest intraspecific distance (Table S1). The interspecific distances ranged from 0.1421 ± 0.0484 to 0.2245 ± 0.0810 (Table S1). The higher level of interspecific distances, compared to intraspecific distances, indicates that the four mantis species can be distinguished based on COI sequence variability.

### 2.2. Development of Species-Specific SCAR Markers and Sensitivity Test

**Figure 1:**
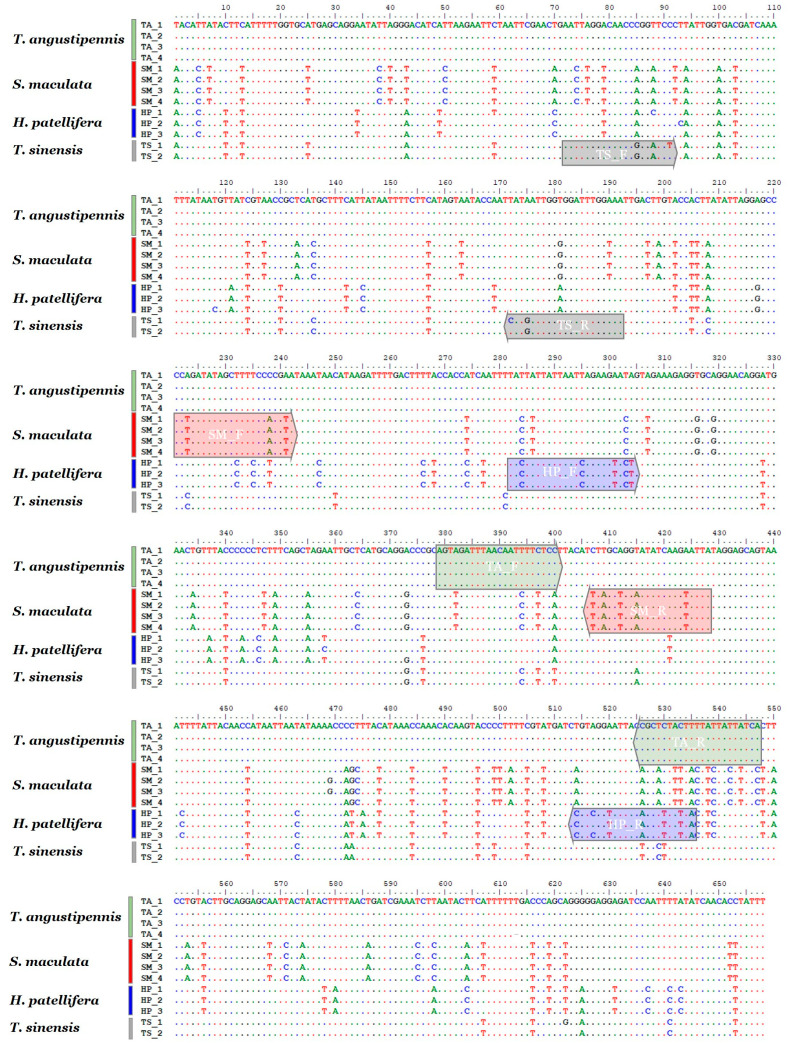
Comparative sequence analysis of the COI regions of the four mantis species. Box-shaped arrows indicate the positions and directions of the four species-specific SCAR primer pairs.

Thirteen COI sequences from the four mantis species were comparatively analyzed, revealing species-specific nucleotide sequences, which were validated for specificity through comparison with sequences from closely related species (Figure S1). Based on these sequences, SCAR primer pairs were designed and optimized to produce PCR amplicons of unique sizes for each species (Figure 1).

**Figure 2:**
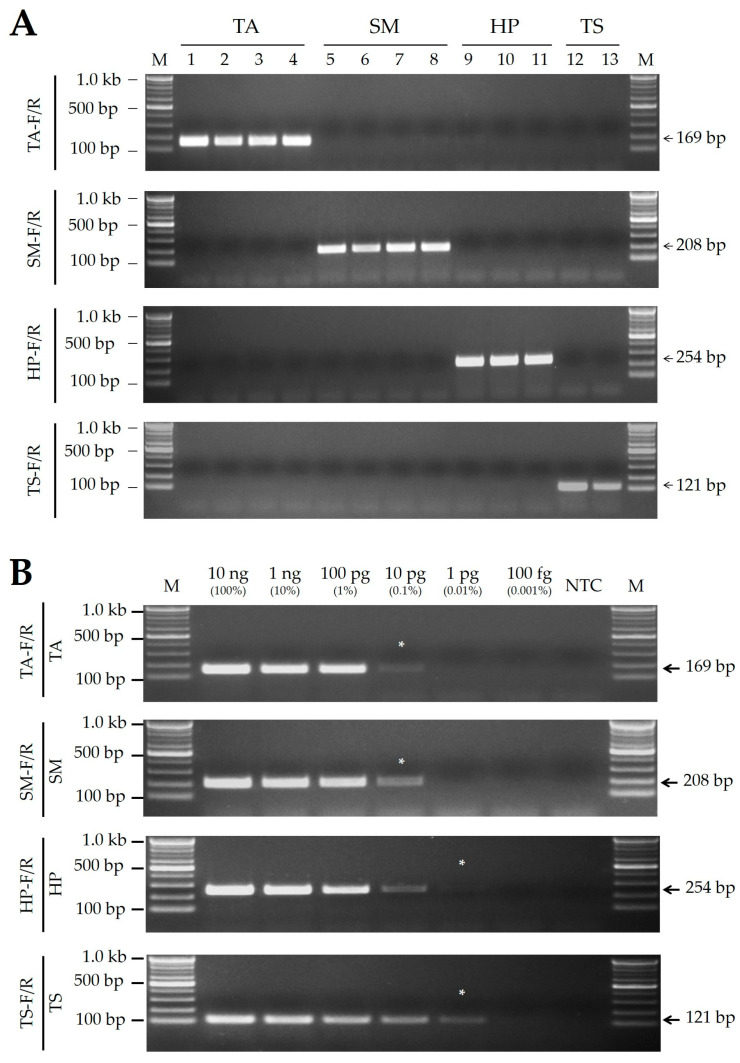
(**A**) PCR amplicons produced using species-specific SCAR markers developed in this study. (**B**) Sensitivity analysis of SCAR markers using serially diluted template DNA. NTC, non-template control; *, minimum detectable DNA concentration for each SCAR marker. The SCAR marker names (TA-F/R, SM-F/R, HP-F/R, and TS-F/R) presented on the left of the gel images correspond to the primer information listed in Table 1, and details of the mantis DNA sample (TA, SM, HP, and TS) are listed in Table 4. Numbers on the right indicate amplicon sizes. M, 100 bp DNA ladder with band sizes as indicated.

Four SCAR primer pairs successfully amplified DNA fragments from the target species, with amplicon sizes of 169, 208, 254, and 121 bp for *T. angustipennis*, *S. maculata*, *H. patellifera*, and *T. sinensis*, respectively (Table 1 and Figure 2A).

The sensitivity of the SCAR markers was examined using serially diluted template DNAs. The results showed that SCAR markers targeting *H. patellifera* and *T. sinensis* had a sensitivity of 0.01%, and those targeting *T. angustipennis* and *S. maculata* had a sensitivity of 0.1% (Figure 2B).

### 2.3. Multiplex SCAR Assay, Discriminability, and Specificity Test

**Figure 3:**
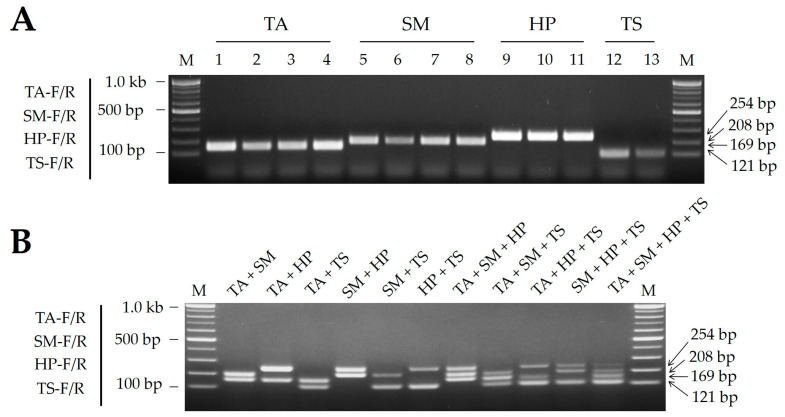
(**A**) PCR amplicons produced using the multiplex SCAR assay developed in this study. (**B**) Discrimination capacity of multiplex SCAR assay. DNA from 2 to 4 mantis species was combined and used as template. All PCRs used all four species-specific primer pairs (TA-F/R, SM-F/R, HP-F/R, and TS-F/R), with the template DNA samples indicated above the gel image. Details of the mantis DNA samples (TA, SM, HP, and TS) are listed in Table 4. M, 100 bp DNA ladder with band sizes as indicated. Numbers on the right indicate amplicon sizes.

The four SCAR primer pairs were then combined into a multiplex SCAR assay capable of distinguishing between all four mantis species in a single PCR. Each primer pair successfully amplified the uniquely sized DNA amplicons relevant to the target species (Figure 3A). The highest-quality multiplex PCR products were generated under optimal PCR conditions for the SCAR primers, as described in Section 2.2.

The discriminatory power of the multiplex SCAR assay was assessed using a DNA mixture of two to four template DNAs from four different mantis species in equal proportions. The multiplex SCAR assay proved to be effective as it successfully amplified each target species. Notably, even when a maximum of four template DNAs was used, the assay could clearly differentiate each amplicon in a single gel electrophoresis column (Figure 3B).

The specificity of the developed SCAR assay was evaluated through the empirical testing of eleven animals, seven plants, and one fungal species. These species are commonly prescribed and dispensed with Mantidis Ootheca in traditional medicine. The SCAR assays generated PCR products of predetermined sizes that corresponded to each target species but did not produce such products for any nontarget species (Table 2, Figure S2, and Table S2). To further clarify, each amplicon was sequenced. The results confirmed that the amplicon sequences matched those of the target species (Figure S3). These findings show that SCAR assays are effective in accurately identifying the targeted mantis species.

### 2.4. Application of SCAR Assays to Commercial Products

**Figure 4:**
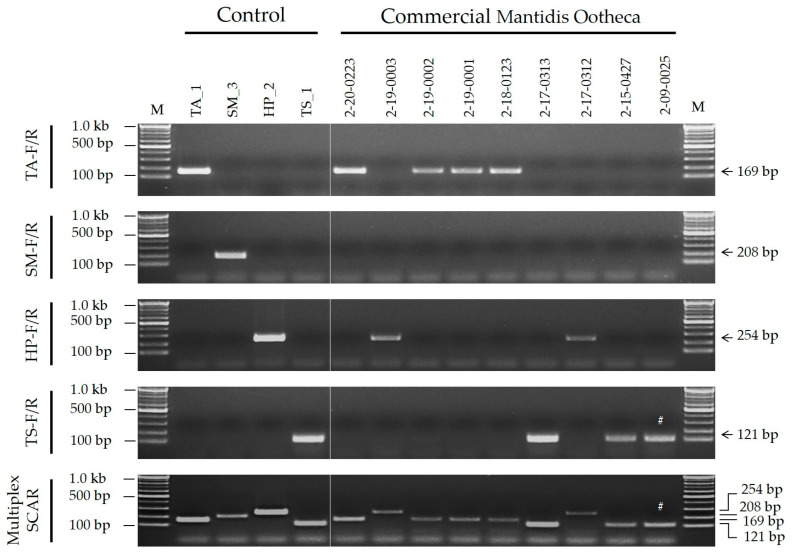
Analysis of commercial Mantidis Ootheca products using both species-specific and multiplex SCAR assays. The SCAR marker names (TA-F/R, SM-F/R, HP-F/R, and TS-F/R) presented on the left of the gel images correspond to the primer information listed in Table 1. Details of control and commercial samples are listed in Table 4 and Table S2, respectively. M, 100 bp DNA ladder with band sizes as indicated; #, counterfeit commercial products according to the country of distribution.

The SCAR assays were validated using commercial samples purchased from various distributors of pharmaceutical materials (including pharmaceutical companies) between 2009 and 2020 (Table S3). Nine commercial Mantidis Ootheca strains were used as the controls. The results revealed the presence of *T. angustipennis* in four samples (2-20-0223, 2-19-0002, 2-19-0001, and 2-18-0123), *H. patellifera* in two samples (2-19-0003 and 2-17-0312), and *T. sinensis* in three samples (2-17-0313, 2-15-0427, and 2-09-0025) (Figure 4). Notably, *S. maculata* was not detected in the commercial samples (Figure 4). Species identification results were identical for both the species-specific and multiplex SCAR assays (Figure 4).

The sources and authenticity of the nine commercial samples were analyzed (Figure 4 and Table S3). Two (2-17-0313 and 2-17-0312) had unknown sources of purchase; one sample was purchased in China (2-15-0427) and was confirmed to be *T. sinensis*, which is an authentic species of Mantidis Ootheca in China; six samples were purchased in Korea, of which five were confirmed to be authentic *T. angustipennis* or *H. patellifera*; and one (2-09-0025) was deemed a counterfeit, *T. sinensis*, which is not authentic in Korea but authentic in China (Figure 4). The outcomes of molecular species identification were compared with those of morphological species identification (Table 3). Eight of the nine samples exhibited congruent molecular and morphological results. One of the commercial samples that had been processed into powder form (2-15-0427) was identified as *T. sinensis* using SCAR assays (Figure 4); however, the morphological identification of this sample was unfeasible (Table 3). Thus, the SCAR assays successfully identified the original species of commercial Mantidis Ootheca, even in powdered samples.

## 3. Discussion

In this study, the PCR discrimination analysis of the commercial samples (Figure 4) revealed that one sample (2-09-0025) contained *T. sinensis*, which is an inauthentic species in Korea. This finding indicates that Mantidis Ootheca commercially distributed in the Korean oriental medicine market may contain inauthentic species. Korea and China have different authentic species of Mantidis Ootheca. As shown in Table S3, distributors of Korean oriental medicine commonly import Mantidis Ootheca from China, where *T. sinensis* is an authentic Mantidis Ootheca species. The difference in the authentic species between Korea and China, coupled with Korea’s high dependency on the import of Mantidis Ootheca from China, is the root cause of the distribution of adulterated commercial products in Korea.

The emergence of counterfeit and adulterated animal-based traditional medicine poses safety risks, creating a significant challenge for traditional medicine trade supervision and quality control. Identifying and authenticating animal species is crucial to ensure the safety and quality of animal-derived medicinal products [34].

In Asian countries including Korea and China, the use of Mantidis Ootheca is a common and valued practice in traditional medicine [16]. The correct species listed in the Pharmacopoeia must be used; however, identifying the species of mantis that produced the Ootheca can be challenging. Several studies have attempted to develop methods for identifying the species of Mantidis Ootheca. Song et al. identified three species of Mantidis Ootheca (*T. angustipennis, T. sinensis*, and *H. patellifera*) based on its external morphology [16]. Using this method, Mantidis Ootheca can be distinguished through the appearance features by people with experience. However, this method has limitations, including potential risks for subjective bias in species identification and the challenge of applying these methods when the sample’s morphology is incomplete [35]. Wang et al. reported research results on identifying Mantidis Ootheca based on DNA barcoding [33]. However, due to the constraints of traditional DNA barcoding techniques, this method is also inconvenient for laboratories studying the efficacy of Mantidis Ootheca or for inspection agencies that need to verify its authenticity. Firstly, when universal primers are used, the DNA of nontarget species, such as storage pests, can also be amplified, making it challenging to obtain precise results [35]. Secondly, when specific primers unique to mantis species are used, there is a reduced likelihood of amplifying storage pests, but this method is still time-consuming and costly because it involves post-PCR processing for sequencing, the sequencing itself, and further processing of the sequence data, such as comparing it with reference sequences before obtaining the final sequence [36].

The SCAR marker developed in this study eliminates the possibility of subjective interference by the identifier, providing objective identification results compared to morphological identification. Moreover, identification is feasible even for samples that are not morphologically intact, as long as DNA can be extracted. Compared with traditional DNA barcoding techniques, the SCAR marker only amplifies species that can be used as materials for Mantidis Ootheca, with the results unaffected by storage pests. Moreover, the use of these SCAR markers enables species identification with only one round of PCR and electrophoresis, thereby reducing the time and cost associated with post-processing and sequencing [37].

In a different study, Xu et al. utilized metabarcoding to identify Mantidis Ootheca species [15]. DNA metabarcoding is also an excellent technique for the accurate identification of species. However, despite recent advances in next-generation sequencing (NGS) and its expanding usage, not all molecular biology laboratories have access to NGS equipment. Even if the task is outsourced to analysis companies, obtaining results still incurs significant time and monetary costs. Therefore, it is challenging for individual laboratories to apply DNA metabarcoding techniques to each sample that requires verification. In contrast, traditional PCR amplifiers are available in most molecular biology laboratories. Consequently, the SCAR marker developed in this study offers the significant advantage of enabling easy and rapid species identification in any laboratory equipped with a PCR amplifier. This advantage allows for its utilization in various applications, such as efficacy studies using Mantidis Ootheca and verifying raw materials for preparing prescriptions containing Mantidis Ootheca.

However, the use of a marker for species identification is limited in cases wherein the target species is absent in an unknown sample, rendering species discrimination unfeasible. Therefore, to enhance the utility of this marker, during the experimental design phase, the target species must be carefully selected from those that are similar in appearance to the original species, share habitats, or have similar common names, which could lead to confusion with the reference species during the collection and distribution processes.

Overall, in the identification of Mantidis Ootheca, establishing a comprehensive identification system that integrates the aforementioned morphological identification methods, DNA barcoding, and SCAR marker assays would be the most effective approach. This integrated assay method would significantly help in accurately distinguishing different forms of Mantidis Ootheca (such as whole, powdered, or fragments) and in standardizing quality.

## 4. Materials and Methods

### 4.1. Materials and Obtaining COI Sequences

A total of thirteen specimen samples from four different mantis species were used for both DNA barcode analysis and SCAR marker development (Table 4). Specimens of *T. angustipennis* (TA_1–TA_3) and *S. maculate* (SM_1–SM_4) were obtained from the Korea National Arboretum (Pocheon, Korea). The bodies of *T. angustipennis* (TA_4), *H. patellifera* (HP_1–HP_3), and *T. sinensis* (TS_1 and TS_2) were collected from urban areas during the summer of 2018 and identified by an insect taxonomy expert (Bong-kyu Byun, a professor at Hannam University, Daejeon, Korea). All samples were stored in 100% ethanol at −20 °C until further analysis. Nine commercial Mantidis Ootheca samples were purchased from various distributors of oriental medicine from 2009 to 2020 and deposited in the Korean Herbarium of Standard Herbal Resources (Index Herbariorum code KIOM) with unique voucher numbers (Table 4). Prior to molecular analysis, commercial Mantidis Oothecae were morphologically identified using the reported diagnostic characteristics [16].

Genomic DNA was extracted from one leg of each mantis following the protocol described by Noh et al. [25]. The concentration of genomic DNA was adjusted to approximately 15 ng/μL; then, it was stored at −20 °C for further analysis. The primers (CO1–C02 5′-AYT CAA CAA ATC ATA AAG ATA TTG G-3′ and CO1–C04 5′-ACY TCR GGR TGA CCA AAA AAT CA-3′) developed by Che et al. were used to amplify a fragment of the mitochondrial COI [38]. The PCR amplifications were carried out in 40 μL reaction volumes containing 10 mmol/L Tris-HCl (pH 9.0), 2.5 mmol/L MgCl_2_, 200 μmol/L of each dNTP, 10 mmol/L (NH_4_)_2_SO_4_, 0.5 U Taq DNA polymerase (Solgent, Daejeon, Korea), 0.5 μmol/L of each primer, and approximately 15 ng of template DNA. The PCR amplification was performed using a DNA Engine Dyad^®^ PTC-0220 (Bio-Rad, Foster City, CA, USA), with thermal conditions as follows: 95 °C for 5 min; 35 cycles of 1 min at 95 °C, 1 min at 45 °C, and 1 min at 72 °C; and a final extension for 5 min at 72 °C [16]. Following amplification, the PCR products were separated using 1.5% agarose gel electrophoresis with a 100 bp DNA ladder (Solgent, Daejeon, Korea) [16]. Proper amplified DNA fragments were cut and retrieved from agarose gels using a QIAquick^®^ Gel Extraction Kit (Qiagen, Valencia, CA, USA) and subcloned into the pGEM-T Easy vector (Promega, Madison, WI, USA). Inserted fragments were sequenced in both directions using an automatic DNA sequence analyzer (ABI 3730, Applied Biosystems Inc., Foster City, CA, USA) [16].

### 4.2. Nucleotide Sequence Analyses and SCAR Marker Development

The obtained COI sequences were manually aligned and edited using ClustalW multiple alignment embedded in the BioEdit program (ver. 7.2.5) [39] and confirmed by the National Center for Biotechnology Information (NCBI) Basic Local Alignment Search Tool (BLAST). The final COI sequences were deposited in GenBank (accession numbers are listed in Table 4). Species-specific nucleotides were detected over the aligned sequences using BioEdit. SCAR primers (21–23 bp) were designed to contain one or more species-specific nucleotides near the 3′ end and to produce uniquely sized PCR amplicons for each species. The PCR mixture was used to assess the operability of each primer combination following the protocol described by Kim et al. [40]. The final optimized PCR conditions for SCAR primers were as follows: initial denaturation at 95 °C for 2 min, followed by 35 cycles of denaturation at 95 °C for 30 s, annealing at 55 °C for 30 s, and extension at 72 °C for 30 s, and a final extension at 72 °C for 5 min. The direction and position of the SCAR primers are shown in Figure 1.

### 4.3. Multiplex SCAR Assay and Market Monitoring

To reduce the time required for molecular species identification, all four species-specific primer pairs were combined into a single PCR mixture for multiplex PCR. The conditions for multiplex PCR were optimized for several PCR parameters, including the annealing temperature and duration, number of PCR cycles, and concentrations of primers and template DNAs. The performance of multiplex PCR amplifications was assessed via agarose gel electrophoresis on a 1.5% agarose gel stained with Ecodye™ Nucleic Acid Staining Solution (Biofact, Daejeon, Korea) with a 100 bp DNA ladder (Solgent, Daejeon, Korea).

## 5. Conclusions

Mantidis Ootheca, the egg case of the mantis, is a traditional medicine derived from insects. In terms of medical use, the quality of Mantidis Oothecae, including the authentication of taxonomic origin, is difficult to verify because of the complex morphological differences. To overcome this challenge, SCAR markers were developed for four mantis species using COI sequences. These markers can help distinguish authentic Mantidis Oothecae from counterfeits. The SCAR markers successfully amplified the expected amplicons from the target species but not from the nontarget species, even in the multiplex SCAR assay and when low-concentration template DNA was used. SCAR markers were also used to evaluate nine commercial Mantidis Ootheca products, of which eight were confirmed to be authentic based on the species defined for each purchased country, whereas one was inauthentic. Thus, the SCAR assay methods established in this study can be used to identify authentic Mantidis Ootheca and help standardize and control its quality in commercial products.

## Figures and Tables

**Table 1:** Primer sequences and amplicon sizes of SCAR markers.

Target Species	Primer	Sequence (5′-3′)	Size (bp)
*Tenodera angustipennis*	TA-F	AGT AGA TTT AAC AAT TTT CTG C	169
	TA-R	TGA TAA TAA TAA AAG TAG AGC G	
*Statilia maculata*	SM-F	CCT GAT ATA GCT TTT CCA CGT	208
	SM-R	TAA TAC TTG ATA TTC CAG CTC A	
*Hierodula patellifera*	HP-F	TAC TAT TAT TAA TCA GAA GTC CT	254
	HP-R	TAA TAA AGC TGT AAT ACC GAC G	
*Tenodera sinensis*	TS-F	AAT TAG GAC AAC CGG GAT CT	121
	TS-R	TTT CCA AAT CCA CCA ATC ACG	

**Table 2:** Specificity of SCAR markers.

Scientific Name	Herbal Name	Species-Specific SCAR				Multiplex SCAR
		TA	SM	HP	TS	
*Tenodera angustipennis*	Mantidis Ootheca	+	-	-	-	+(TA)
*Statilia maculata*	Mantidis Ootheca	-	+	-	-	+(SM)
*Hierodula patellifera*	Mantidis Ootheca	-	-	+	-	+(HP)
*Tenodera sinensis*	Mantidis Oötheca	-	-	-	+	+(TS)
*Scolopendra subspinipes*	Scolopendra	-	-	-	-	-
*Metaphire guillelmi*	Pheretima	-	-	-	-	-
*Cryptotympana atrata*	Cicadidae Periostracum	-	-	-	-	-
*Bombyx mori*	Batryticatus Bombyx	-	-	-	-	-
*Pelodiscus sinensis*	Pelodiscis Carapax	-	-	-	-	-
*Hippocampus trimaculatus*	Hippocampus	-	-	-	-	-
*Elaphe carinata*	Serpentis Periostracum	-	-	-	-	-
*Schisandra chinensis*	Schisandrae Fructus	-	-	-	-	-
*Zanthoxylum schinifolium*	Zanthoxyli Pericarpium	-	-	-	-	-
*Aralia continentalis*	Araliae Continentalis Radix	-	-	-	-	-
*Cynanchum wilfordii*	Cynanchi Wilfordii Radix	-	-	-	-	-
*Sigesbeckia glabrescens*	Siegesbeckiae Herba	-	-	-	-	-
*Rheum rhabarbarum*	Rhei Undulatai Rhizoma	-	-	-	-	-
*Machilus thunbergii*	Machilii thunbergii cortex	-	-	-	-	-
*Ophiocordyceps sinensis*	Cordyceps	-	-	-	-	-

**Table 3:** Comparison of the results of molecular discrimination using SCAR markers and the results of morphological discrimination.

Type	Voucher Number	Identified Species by Morphology	Species-Specific SCAR				Multiplex SCAR
			TA	SM	HP	TS	
Dried whole	2-20-0223	*T. angustipennis*	+	-	-	-	+(TA)
Dried whole	2-19-0003	*H. patellifera*	-	-	+	-	+(HP)
Dried whole	2-19-0002	*T. angustipennis*	+	-	-	-	+(TA)
Dried whole	2-19-0001	*T. angustipennis*	+	-	-	-	+(TA)
Dried whole	2-18-0123	*T. angustipennis*	+	-	-	-	+(TA)
Dried whole	2-17-0313	*T. sinensis*	-	-	-	+	+(TS)
Dried whole	2-17-0312	*H. patellifera*	-	-	+	-	+(HP)
Powder	2-15-0427	Unknown	-	-	-	+	+(TS)
Dried whole	2-09-0025	*T. sinensis*	-	-	-	+	+(TS)

**Table 4:** Materials used in this study.

No.	Scientific Name	Herbal Name		Voucher Number	Collection Site	Collection Date	NCBI Accession	Abbreviation
		KHP	ChP					
1	*Tenodera angustipennis*	Mantidis Ootheca	-	KNAE455837	Ongjin, Incheon, Korea	15 September 2014	OL913116	TA_1 *
2				KNAE410919	Jeju, Jeju-do, Korea	29 August 2013	OL913117	TA_2
3				KNAE451914	Jeju, Jeju-do, Korea	29 August 2013	OL913118	TA_3
4				KIOM2018-11	Yuseong, Daejeon, Korea	24 August 2018	OL913119	TA_4
5	*Statilia maculata*		Mantidis Oötheca	KNAE410802	Jeju, Jeju-do, Korea	29 August 2013	OL913120	SM_1
6				KNAE451475	Ongjin, Incheon, Korea	16 September 2014	OL913121	SM_2
7				KNAE510527	Pocheon, Gyeonggi-do, Korea	30 September 2014	OL913122	SM_3 *
8				KNAE455907	Ongjin, Incheon, Korea	15 September 2014	OL913123	SM_4
9	*Hierodula patellifera*			KIOM2018-02	Yuseong, Daejeon, Korea	23 August 2018	OL913124	HP_1
10				KIOM2018-04	Yuseong, Daejeon, Korea	23 August 2018	OL913125	HP_2 *
11				KIOM2018-09	Yuseong, Daejeon, Korea	24 August 2018	OL913126	HP_3
12	*Tenodera sinensis*	-		KIOM2018-07	Yuseong, Daejeon, Korea	23 August 2018	OL913127	TS_1 *
13				KIOM2018-10	Yuseong, Daejeon, Korea	24 August 2018	OL913128	TS_2

KHP, The Korean Herbal Pharmacopoeia, 2020. (Republic of Korea); ChP, Pharmacopoeia of the People’s Republic of China 2015 edition, 2015. (China); * Individual samples used to test the sensitivity of SCAR markers (Figure 2B) and the discrimination capacity of multiplex-SCAR assay (Figure 3B) and as controls for analysis of commercial preparations (Figure 4).

## Data Availability

The original contributions presented in the study are included in the article and Supplementary Materials; further inquiries can be directed to the corresponding author.

## Supplementary Materials

The following supporting information can be downloaded at https://www.mdpi.com/article/10.3390/ijms251810224/s1.
