# Supplementary material for: Rapid Diagnostic PCR Assay Method for Species Identification of Mantidis Ootheca (Sangpiaoxiao) Based on Cytochrom C Oxidase I (COI) Barcode Analysis
Source: Int J Mol Sci. 2024 Sep 23;25(18):10224. doi: 10.3390/ijms251810224 (PMC11432583; doi:10.3390/ijms251810224)
Supplement: Supplementary file 1 [file ijms-25-10224-s001.zip › ijms-3180533-supplementary.pdf]

Supplementary Material

Rapid diagnostic PCR assay for species identification of Mantidis Ootheca (Sangpiaoxiao) based on COI barcode analysis

Sumin Noh, Wook Jin Kim, Ji-Min Cha, Goya Choi, Sungyu Yang, Jun-Ho Song and Byeong Cheol Moon*

Table S1. Characteristics of the COI region in the four mantis species.

Scientific name	Sequence length (bp)	Intra-specific distance	Inter-specific distance
Tenodera angustipennis	658	0.0000 ± 0.0000	0.1888 ± 0.0644
Statilia maculata	658	0.0010 ± 0.0010	0.2245 ± 0.0810
Hierodula patellifera	658	0.0072 ± 0.0033	0.1989 ± 0.0739
T. sinensis	658	0.0046 ± 0.0031	0.1421 ± 0.0484

Table S2. Samples used for specificity analysis of the SCAR markers developed in this study.

No.	Scientific name	Voucher number	Organism	Gel lane
1	*T. angustipennis*	KNAE455837	Animalia	TA_1
2	*S. maculata*	KNAE510527		SM_3
3	*H. patellifera*	KIOM2018-04		HP_2
4	*T. sinensis*	KIOM2018-07		TS_1
5	*Scolopendra subspinipes*	2-18-0113		SS
6	*Metaphire guillelmi*	2-18-0118		MG
7	*Cryptotympana atrata*	2-18-0119		CA
8	*Bombyx mori*	2-18-0121		BM
9	*Pelodiscus sinensis*	2-14-0119		PS
10	*Hippocampus trimaculatus*	2-12-0027		HT
11	*Elaphe carinata*	2-17-0653		EC
12	*Schisandra chinensis*	2-19-0429	Plantae	SC
13	*Zanthoxylum schinifolium*	2-19-0421		ZS
14	*Aralia continentalis*	2-19-0413		AC
15	*Cynanchum wilfordii*	2-19-0417		CW
16	*Sigesbeckia glabrescens*	2-19-0437		SG
17	*Rheum rhabarbarum*	2-19-0432		RR
18	*Machilus thunbergii*	2-19-0445		MT
19	*Ophiocordyceps sinensis*	2-2016-F027	Fungi	OS

Table S3. List of Mantidis Ootheca samples purchased from oriental medicine markets used to verify the newly developed SCAR marker assay.

Voucher number	Distributor (Purchase Country)	Country of Origin	Purchase (donated) Year
2-20-0223	G○○ pharmaceutical company (South Korea)	China	2020
2-19-0003	U○○ pharmaceutical company (South Korea)	China	2019
2-19-0002	M○○ pharmaceutical company (South Korea)	China	2019
2-19-0001	G○○ pharmaceutical company (South Korea)	China	2019
2-18-0123	G○○ pharmaceutical company (South Korea)	China	2018
2-17-0313	Unknown-donated	Unknown	2017
2-17-0312	Unknown-donated	Unknown	2017
2-15-0427	S○○ pharmaceutical company (China)	China	2015
2-09-0025	D○○ pharmaceutical company (South Korea)	China	2009

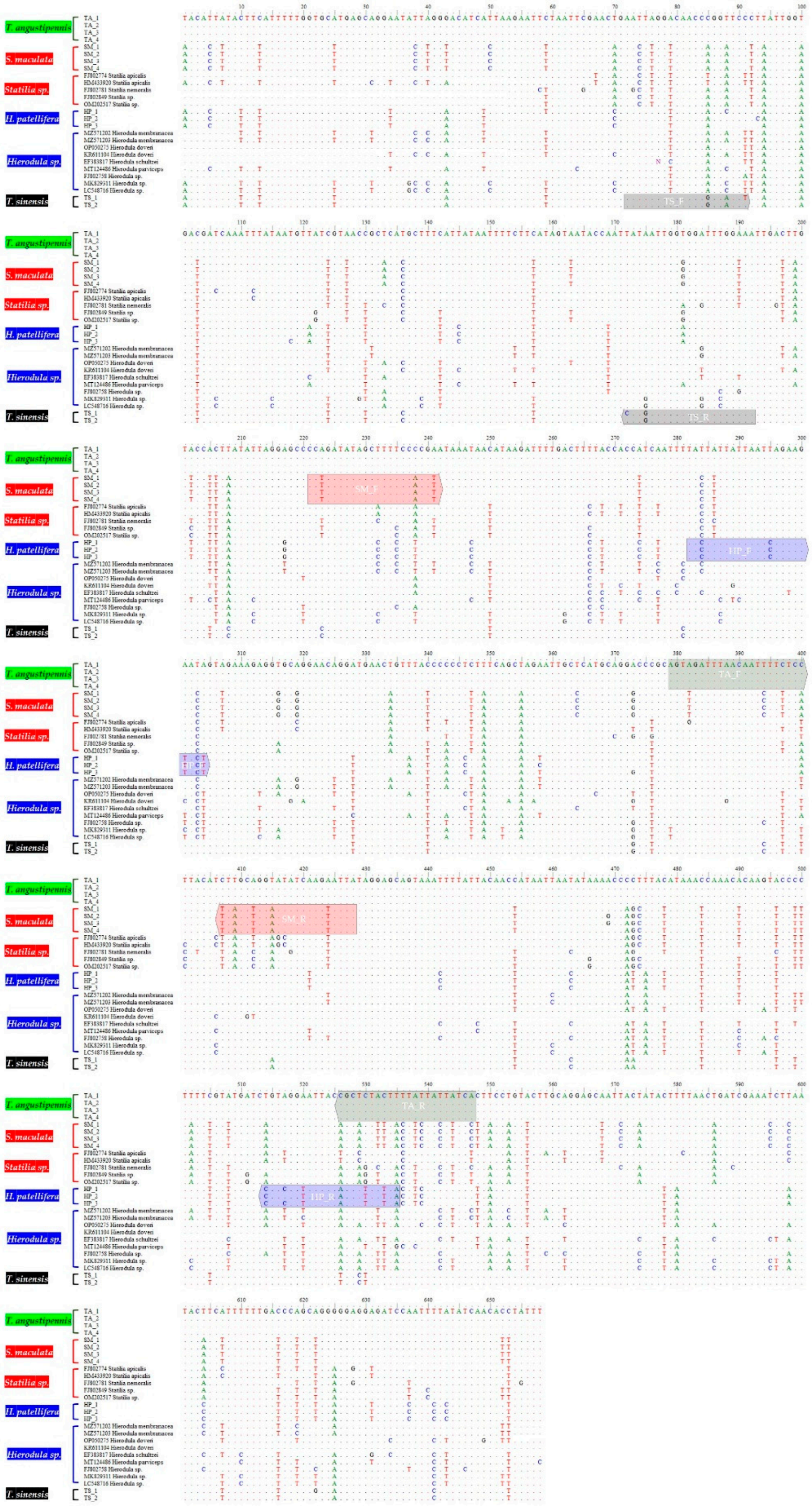

Figure S1. ClustalW multiple alignment of cytochrome c oxidase I (COI) sequences among 12 species, including closely related with four species; *T. angustipennis*, *S. maculata*, *H. patellifera*, and *T. sinensis*. Colored boxes indicate species-specific primers. Dots (•) indicate the identical sequences with TA_1 and dashes (-) represent gaps introduced to maximize alignment.

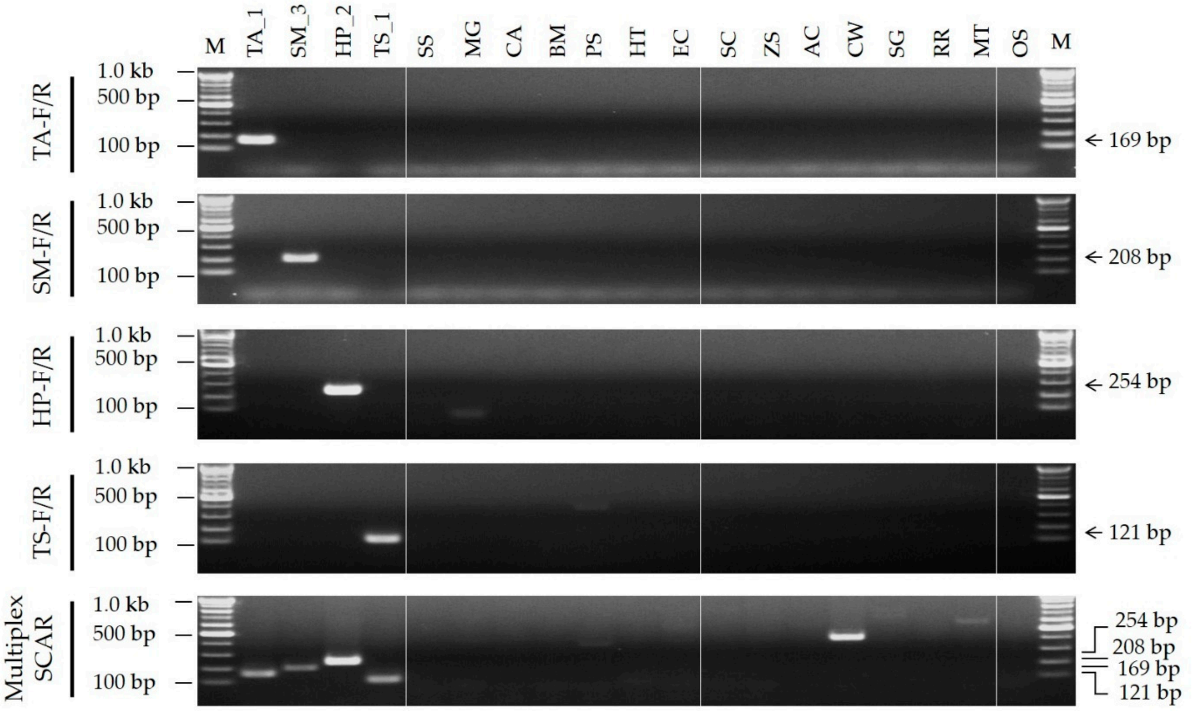

Figure S2. Specificity of the SCAR markers. The SCAR marker names (TA-F/R, SM-F/R, HP-F/R, and TS-F/R) presented on the left of the gel images correspond to the primer information listed in Table 1. Details of the control and commercial samples are listed in Tables S1 and S4, respectively. M, 100 bp DNA ladder with band sizes as indicated.

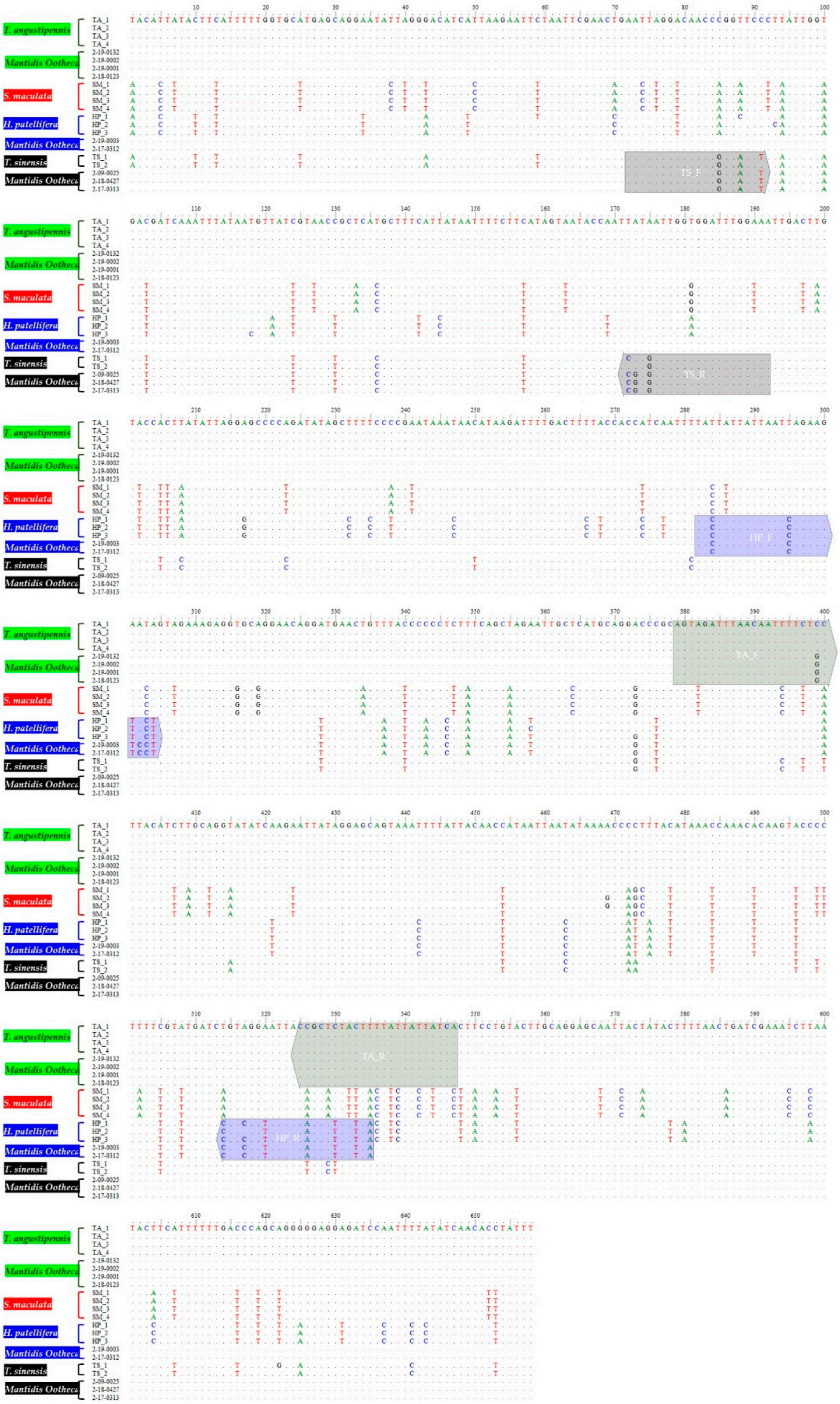

Figure S3. Species specificity confirmation by sequence analysis of SCAR amplicons. Colored boxes indicate species-specific primers. Dots (•) indicate the identical sequences with TA_1 and dashes (-) represent gaps introduced to maximize alignment.
